# Effects of yoga on stress in stressed adults: a systematic review and meta-analysis

**DOI:** 10.3389/fpsyt.2024.1437902

**Published:** 2024-11-01

**Authors:** Alina Schleinzer, Alina Moosburner, Dennis Anheyer, Laura Burgahn, Holger Cramer

**Affiliations:** ^1^ Institute for General Practice and Interprofessional Care, University Hospital Tübingen, Tübingen, Germany; ^2^ Robert Bosch Centre for Integrative Medicine and Health, Bosch Health Campus, Stuttgart, Germany; ^3^ Department of Psychology and Psychotherapy, University Witten/Herdecke, Witten, Germany

**Keywords:** stress, yoga, complementary medicine, meta-analysis, systematic review

## Abstract

**Background:**

With the increasing prevalence of stress, stress reduction is getting more and more important. Yoga is being considered as a non-pharmacological treatment option for stress.

**Objective:**

Investigation of the effects of yoga on stress in stressed adults from the general population.

**Methods:**

Databases were searched up to March 17, 2023 (updated search on May 17, 2024). Randomised controlled trials (RCTs) of yoga for stressed adults were included if they assessed perceived stress. Further outcomes were quality of life and stress-related physiological measures. Standardised mean differences (SMDs) and 95% confidence intervals (CIs) were calculated. The quality of the included studies was assessed using the Cochrane tool, and the quality of the evidence for each outcome was graded according to the GRADE recommendations.

**Results:**

A total of 13 RCTs with 1026 participants were included in the qualitative analysis and nine RCTs in the quantitative analysis. The meta-analyses revealed low quality of evidence for short-term effects of yoga on stress compared to passive control groups (SMD=-0.69, 95%CI=-1.12- -0.25) and low quality of evidence for long-term effects on stress in favour of active control groups (SMD=0.23, 95%CI=0.06-0.40). There was moderate quality of evidence for short-term effects of yoga on quality of life compared to passive control groups (SMD=0.86, 95%CI=0.72-1.00). No adverse events occurred in the three safety reporting studies.

**Conclusions:**

As there is low quality of evidence for short-term effects of yoga on perceived stress in stressed adults from the general population compared to passive control groups, yoga can be considered as a non-pharmacological treatment option for stress reduction.

**Systematic review registration:**

https://inplasy.com/wp-content/uploads/2023/03/INPLASY-Protocol-4595.pdf INPLASY, identifier 202330062.

## 1 Introduction

Recent data showed a marked increase in the prevalence of stress compared with the pre-pandemic situation (1). While stress is a natural response to potential threats in the environment (2) and to some extent necessary to cope with the challenges of daily life, too much or too prolonged stress can lead to health problems (3, 4) such as cardiovascular diseases (5), chronic headaches (6), depression and anxiety (7) and is associated with increased mortality (8). There are also social and economic consequences of stress. For example, stressed individuals often tend to “retract from social interactions and tend to be irritable and hostile” (9). They are less productive at work (10) and increase turnover rates in the company (11), which not only affects companies in monetary terms, but also the social costs of the country (12).

Given the growing prevalence of stress and its far-reaching consequences, methods for stress reduction are becoming increasingly important. One such non-pharmacological treatment option for stress reduction is yoga (13). Yoga is an increasingly popular therapeutic intervention and is the most widely used intervention in complementary medicine (14). Complementary medicine includes interventions and treatments “that are used along with standard treatments, but are not considered standard” (15). Yoga originated in Indian philosophy and involves physical postures, ethical lifestyle and spiritual practice to bring body, mind and spirit into harmony (16). Yoga offers a wide variety of types and styles (7), ranging from gentle meditative practises to more physically demanding forms, that can be used depending on the target group and their needs and abilities. Additionally, yoga can be personalised by adjusting the intensity, duration or techniques (17). In Europe and North America yoga mainly includes physical postures (asana) as well as breathing techniques (pranayama) and meditation (dhyana) (16). Yoga can be practiced in yoga classes, at home or anywhere else without the need to buy any yoga equipment (13, 14). However, the time required for yoga practice can be a barrier for some people (13). Consequently, time is a key factor influencing the accessibility and utilisation of non-pharmacological interventions such as yoga.

One of the key benefits of yoga is that it reduces the levels of the stress hormone cortisol, as well as lowering blood pressure and heart rate. Additionally, yoga activates the parasympathetic nervous system and promotes relaxation (18). Controlled breathing techniques, which are central to yoga, increase the oxygen supply to the body, which supports muscle relaxation and helps release tension (17). Furthermore, yoga promotes mindfulness, increasing awareness of negative thoughts so that they can be recognised and managed more effective (19). Research has shown that yoga has beneficial effects on a range of stress-related physiological and psychological conditions, including depression and anxiety (7), low back pain (20), hypertension (21) and diabetes mellitus type 2 (22). This raises the question of whether yoga should not be practised to reduce stress itself and thus prevent the onset of various stress-related diseases.

For this reason, and in line with the fact that yoga is commonly used to reduce health-related stress (14), studies have investigated the effect of yoga on perceived stress and found a positive effect. In a 2011 systematic review of eight studies, Chong et al. reported that yoga had beneficial effects on stress in healthy adults (23). A recent systematic review of six studies examined the effect of yoga on work-related stress and found that yoga had a significant effect on work-related stress (24). To date, no systematic review has examined the effects of yoga on stress in stressed adults in the general population. Therefore, the purpose of this systematic review and meta-analysis is to summarise the current evidence on the effects of yoga on stress reduction in stressed adults in the general population.

## 2 Methods

The review was conducted according to the Preferred Reporting Items for Systematic Reviews and Meta-Analyses guidelines (PRISMA) (25) and the recommendations of the Cochrane Handbook (26). This review was prospectively registered on the International Platform of Registered Systematic Review and Meta-analysis Protocols (INPLASY) under the registration number: 202330062.

### 2.1 Literature search

The electronic databases Medline/PubMed, Cochrane Library, Scopus, PsycINFO and BASE were searched up to March 17, 2023. Medical Subject Headings (MeSH) or equivalent text terms were used around the search terms ‘yoga’ and ‘stress’. Searches were adapted for each database. For Medline, the following search strategy was used: (“Yoga”[MeSH] OR yoga*[Title/Abstract] OR yogi*[Title/Abstract] OR asana*[Title/Abstract] OR pranayama [Title/Abstract] OR dhyana [Title/Abstract] OR dharana [Title/Abstract] OR “Surya Namaskar*” [Title/Abstract]) AND (stress*[Title/Abstract] OR “Stress, Psychological”[MeSH] OR “Stress, Physiological”[MeSH] OR “Occupational Stress”[MeSH] OR “Psychological Distress” MeSH] OR “Financial Stress”[MeSH]). There were no language restrictions for eligibility. Two review authors (AS, LB) independently screened and selected titles and abstracts identified by the literature search, read potentially eligible articles in full and assessed whether they met the inclusion criteria. Disagreements about the inclusion of articles were discussed with a third reviewer (HC) until consensus was reached. Due to the length of the review process, the search and screening was updated on May 17, 2024. No further studies were found that met the inclusion criteria.

### 2.2 Eligibility criteria

Eligibility criteria were defined according to the PICOS (Population, Intervention, Comparison, Outcome measure, Study type) scheme.

1. Types of participants: Studies were eligible if they included adults (>18 years), defined as healthy and/or part of the general population, who self-reported being stressed (reported symptoms of stress or had higher levels of stress according to the questionnaire used) not related to a medical condition. There were no gender restrictions.
2. Types of interventions: The intervention could be any form of yoga (i.e. Hatha yoga, Ashtanga yoga, Iyengar yoga, yoga therapy or any other form of yoga). Studies were also eligible if they did not mention a specific form of yoga, but simply described the intervention as ‘yoga’. Studies were excluded if yoga was not the main intervention, but was part of a multimodal intervention.
3. Types of comparisons: Studies were eligible if they compared yoga to passive controls or any active control.
4. Types of outcome measures: Studies were included if they assessed the primary outcome of this systematic review, stress, as self-reported stress levels using common scales. The secondary outcomes of this systematic review were health-related quality of life, stress-related physiological measures and safety data, including adverse events.
5. Types of studies: Studies were eligible if they were randomised controlled trials (RCTs). There were no restrictions on the type of publication.

### 2.3 Data extraction and management

Three review authors, working independently in pairs of two review authors each (AS & LB; AS & AM), extracted data from the included studies. Study characteristics regarding setting (e.g. type of study, country of origin), population (e.g. age and sex), intervention and control conditions (e.g. type, frequency, duration), outcome measures, and safety were extracted. Disagreements in the extracted data were reviewed by a fourth reviewer (HC) and discussed, until consensus was reached.

#### 2.3.1 Risk of bias in individual studies

The risk of bias for each study was assessed independently by two reviewers (AS, AM). The tool used was the Cochrane Risk of Bias 2 tool (27), which assesses the following five domains: randomisation process, deviations from the intended interventions, missing outcome data, measurement of the outcome and selection of the reported outcome. Each domain was rated as low risk, some concerns or high risk. No overall risk of bias was calculated. Disagreements in the assessment of risk of bias in individual studies were discussed with a third review author (DA) until consensus was reached.

#### 2.3.2 Rating of quality of evidence

According to the Grading of Recommendations Assessment, Development and Evaluation (GRADE) working group, the quality of the evidence for yoga for stress reduction in stressed adults was rated independently by two reviewers (AS, AM) as high, moderate, low or very low (28). Disagreements about the quality of evidence for each outcome were discussed with a third review author (HC) until consensus was reached.

### 2.4 Data analysis

Meta-analyses were performed separately for trials with active and passive control groups. Analyses were also conducted separately for short-term and long-term follow-ups, with short-term follow-up defined as immediately after the intervention and long-term follow-up defined as measures taken closest to four months after randomisation.

#### 2.4.1 Assessment of overall effect size

If at least two studies were available for a particular outcome, pooled analyses were performed using R software version 4.3.1 (29) along with the “meta” package (30). For continuous outcomes, standardised mean differences (SMDs) along with 95% confidence intervals (CIs) were computed. This involved determining the difference in means between groups and dividing it by the pooled standard deviation, employing Hedges correction for small study samples. In cases where standard deviations were not directly provided, they were derived from standard errors, confidence intervals, or t-values. Negative SMDs were indicative of favourable effects for the yoga group in terms of stress in comparison to other groups. Conversely, positive SMDs indicated beneficial effects of the yoga intervention on quality of life in contrast to the comparison interventions. When necessary, scores were adjusted by subtracting the mean from the maximum instrument score (31). Random-effects models were employed using the generic inverse variance method. Additionally, the Hartung-Knapp small-sample correction was applied to account for uncertainty in pooling treatment effects from a limited number of heterogeneous studies (32–35). Cohen’s categories were employed to assess the extent of the overall effect size: (1) SMD 0.2 to 0.49: small; (2) SMD 0.5 to 0.8: moderate; and (3) SMD > 0.8: large effect sizes (36).

#### 2.4.2 Assessment of heterogeneity

Statistical heterogeneity between studies was explored using the I^2^ and τ^2^ statistics. I^2^ represents the percentage of variability in treatment estimates, while τ^2^ describes underlying variability between studies. Unlike I^2^, τ^2^ is not systematically influenced by the number of studies or sample size. Interpretation of I^2^ is as follows: 0% to 24% indicates possibly insignificant heterogeneity, 25% to 49% indicates moderate heterogeneity, 50% to 74% indicates substantial heterogeneity, and 75% to 100% indicates considerable heterogeneity (37). For τ^2^ statistics, the restricted maximum-likelihood estimator was employed (31).

#### 2.4.3 Subgroup and sensitivity analyses

Subgroup analyses were performed when relevant subgroups could be identified. To test the robustness of statistically significant results, sensitivity analyses were conducted for studies with a low risk of bias in the domains of the Risk of Bias 2 tool.

#### 2.4.4 Risk of bias across studies

In cases where at least ten studies were included in the meta-analysis, publication bias was assessed by visual inspection of the funnel plots. Roughly symmetric funnel plots indicated a low risk, whereas asymmetrical funnel plots indicated a higher risk of publication bias. In addition, a linear regression test (Egger test) was performed to assess publication bias.

## 3 Results

### 3.1 Literature search

**Figure 1:**
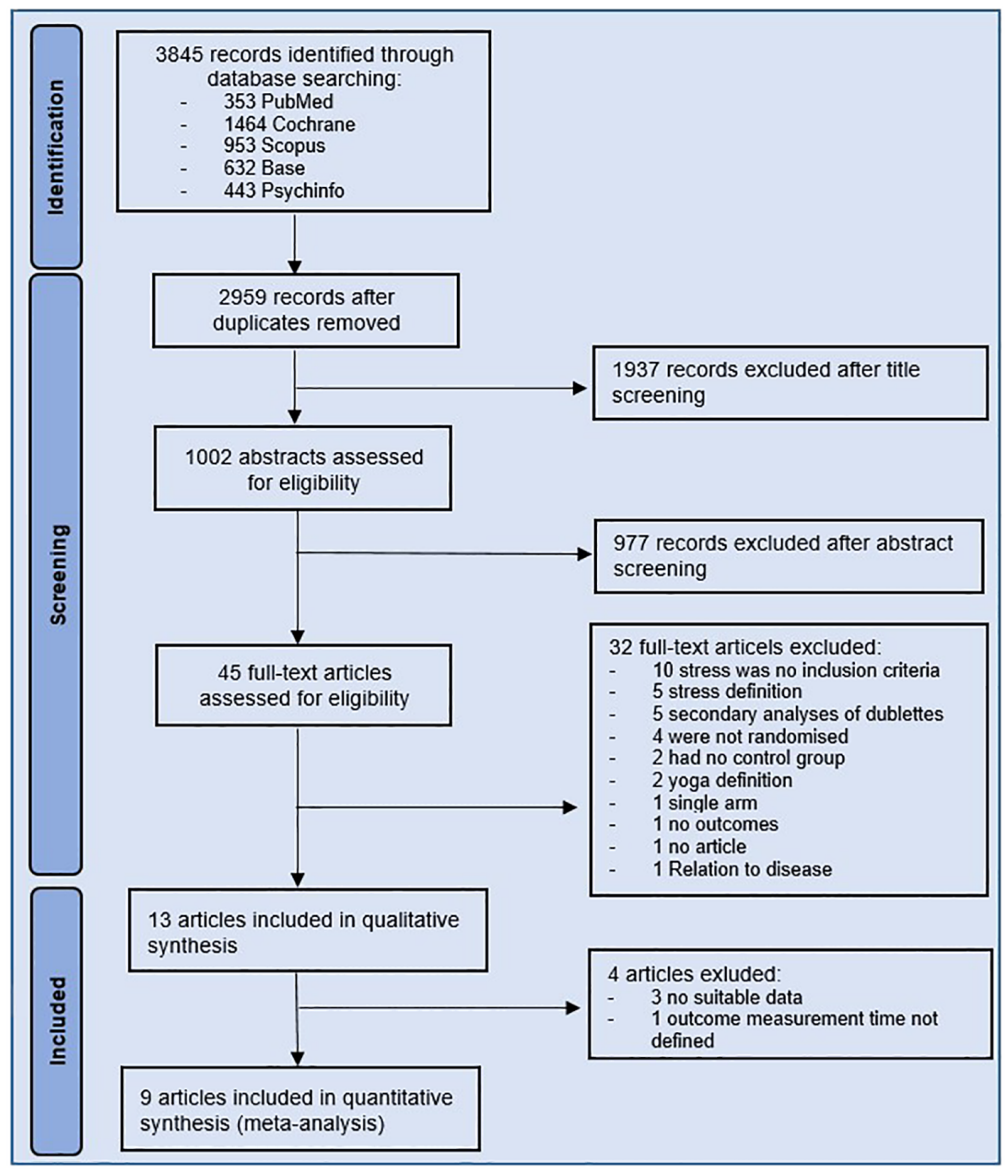
Study flow diagram.

A total of 3845 records were identified through the literature search. Of these, 886 were duplicates, leaving 2959 records for title and abstract screening. After title and abstract screening, 45 potentially eligible studies remained for full text evaluation. Thirty-two studies did not fulfil the inclusion criteria. The reasons for exclusion are shown in **Figure 1**. The remaining 13 studies were included in the systematic review. Four studies of the 13 studies were not included in the meta-analysis, because they did not report adequate outcome data (38–40) or did not define the time of outcome measurement (41). A total of nine studies were included in the meta-analysis.

### 3.2 Study characteristics

#### 3.2.1 Setting and participant characteristics

Study characteristics regarding setting, participants and outcome measures of the 13 included studies are shown in **Table 1**. Included studies were conducted between 2007 and 2022. One study was part of a thesis (39) and the other studies were published in peer-reviewed journals. Of the 13 included studies, four were from Sweden (41–44), three from Germany (13, 39, 49), two each from Australia (46, 47) and the United States (40, 48), and one study each originated from the United Kingdom (45) and Brazil (38). The 13 studies included 1026 participants with mean ages ranging from 33.5 to 55.5 years (median: 45 years). Between 73% and 100% of the participants in each study were female (median: 91.5%). Participants were recruited from the general population (13, 38, 39, 42, 47, 49), companies (40, 41, 45), a university (44) and from a primary health care centre (43). Four studies included working adults (40–45), with one study referring to employees working at home during the pandemic (40). Three studies included only adult women (13, 39, 48), of which one included only adult women at risk of obesity-related diseases (48). One study included sedentary adults (46) and four studies did not further specify the adult participants (42, 43, 47, 49). Common to all the studies was that the participants perceived themselves to be stressed. Thus, the presence of stress as an inclusion criterion was assessed in eight studies by reaching a certain stress score on a stress scale (38, 40, 42, 44–48), in two studies by reporting stress-related symptoms (41, 43) and in three studies by a combination of both (13, 39, 49).

**Table 1:** Study and outcome characteristics.

Author, year	Origin	Recruited from	Inclusion criteria regarding stress	Mean age (SD)	female (in %)	Sample size IG	Sample size CG	Outcome 1.) Stress, 2.) QoL, 3.) stress-related phys. measures	Post treatment assessment points a. short term follow-up; b. long term follow-up	Results: significant group-differences	Safety
Danucalov et al., 2013 (38)	Brazil	general population	at least the resistance stage of stress on LSSI	IG: 55.5 (8.1) CG: 53.4 (8.2)	89.1	25	21	1.) LSSI 3.) Salivary cortisol level	a. Week 8	a. 1.) Yoga > Control	NR
Daukantaite et al., 2018 (42)	Sweden	general population	score of >7 [on a range of 0 to 16] on the four selected items from the PSS	53.5 (6.7)	78.0	A: 34, B: 33	30	1.) PSS-10 3.) Adrenomedullin	a. Week 6	a. 3.) YOMI > Control	NR
Fischer et al., 2022 (49)	Germany	general population	score of >3 [on a range of 0 to 10] on an numeric analog scale for more than one month and at least three stress-related symptoms	46.7 (11.5)	89.2	A: 33, B: 35	34	1.) PSS-10 2.) SF-36	a. Week 12, b. Week 24	NR	A: 0/33 B: 0/35 CG: 0/34
Granath et al., 2007 (41)	Sweden	employees of a company in the financial sector	self-reported stress-related symptoms	NR (-)	73.0	18	19	1.) PSS-14, Daily Stressors 2.) QOLI 3.) Salivary cortisol level, heart rate, blood pressure, urinary catecholamine	unclear	NR	NR
Hewett et al., 2018 (46)	Australia	NR	score of >14 on the stress component of the DASS-21	37.2 (10.8)	79.0	29	34	1.) PSS-10 2.) SF-36	a. Week 8, b. Week 17	a. 1.) Yoga > Control a. 2.) Yoga > Control	NR
Hopkins et al., 2016 (48)	USA	NR	score of >0.4 on PSQ	33.5 (6.4)	100.0	27	25	1.) PSQ 3.) Salivary cortisol level	a. Week 9	a. 1.) Yoga > Control a. 3.) Yoga > Control^a^	NR
Köhn et al., 2013 (43)	Sweden	patients of a primary health care centre	self-reported symptoms of stress and a stress-related diagnosis	53.0 (12)	91.9	18	19	1.) PSS-14 2.) EQ-VAS 3.) Heart rate, blood pressure	a. Week 12	a. 1.) Yoga > Control, a. 2.) Yoga > Control	NR
Maddux et al., 2017 (44)	Sweden	employees of a university	score of >7 [on a range of 0 to 16] on the four selected items from the PSS	46.0 (10)	82.5	43	37	1.) PSS-10 2.) Life satisfaction, HILS	a. Week 8, b. Week 16	a. 1.) Yoga > Control a. 2.) Yoga > Control *(HILS)*	NR
Michalsen et al., 2012 (13)	Germany	general population	score of >18 on the PSS and at least three stress-related symptoms	39.6 (8.3)	100.0	A: 24, B: 24	24	1.) PSS-14 2.) SF-36	a. Week 12	a. 1.) moderate Yoga > Control, intensive Yoga > Control, both Yoga > Control a. 2.) moderate Yoga > Control, intensive Yoga > Control, both Yoga > Control	A: 0/24 B: 0/24 CG: 0/24
Smith et al., 2007 (47)	Australia	general population	mild or moderate levels of stress determined by the GHQ-12	44 (11.8)	83.0	68	63	1.) GHQ-12 2.) SF-36 3.) Blood pressure	a. Week 10, b. Week 16	a 2.) Yoga > Control b 2.) Yoga < Control	NR
Vardar-Wloka, 2019 (39)	Germany	general population	score of >18 on the PSS and at least three stress-related symptoms	NR (-)	100.0	A: 12, B: 12	12	1.) PSS-14 3.) Salivary cortisol level, blood pressure, heart rate, heart rate variability	a. Week 12	NR	A: 0/12 B: 0/12 CG: 0/12
Wadhen & Cartwright, 2021 (45)	UK	employees working from home	score of >5 [on a range of 0 to 10] on an numeric analog scale	42.5 (-)	91.1	17	17	1.) PSS-14, DASS-21 2.) WEMWBS	a. Week 6	a. 2.) Yoga > Control	NR
Wolever et al., 2012 (40)	USA	employees of a company	score of >15 on the PSS-10	42.9 (-)	76.6	90	A: 96, B: 53	1.) PSS-10 3.) Blood pressure, heart rate variability, breathing rate	a. Week 12-14	a. 1.) Yoga > Control B a. 3.) Yoga > Control B *(heart rhythm coherence, breathing rate)*	NR

CG, control group; DASS-21, Depression, Anxiety and Stress Scale - 21 Items; EQ-VAS, Euro Quality of Life Visual Analogue Scale 0;100; GHQ-12, General Health Questionnaire 12-Items; HILS, The Harmony in Life Scale; IG, intervention group; LSSI, Lipp’s Stress Symptoms Inventory for Adults; NR, not reported; PPS, Perceived Stress Scale; PSQ, Perceived Stress Questionnaire; PSS-10, Perceived Stress Scale 10-Items; PSS-14, Perceived Stress Scale 14-Items; QoL, Quality of Life; QOLI, Quality of Life Inventory; SD, standard deviation; sign., significant; UK, United Kingdom; USA, United States of America; WEMWBS, Warwick-Edinburgh Mental Wellbeing Scale; > = favourable significant effects for the yoga group compared to the control group, < = favourable significant effects for the control group compared to the yoga group, ^a^ = only emerged among women who evidenced elevated cortisol reactivity to stress at the baseline.

#### 3.2.2 Interventions characteristics

The characteristics of the interventions are shown in **Table 2**.

**Table 2:** intervention characteristics.

Author, year	Yoga style	Postures	Breathing	Meditation	Duration (in weeks)	Frequency (weekly)	Session duration (in min)	Homework	Control group
Danucalov et al., 2013 (38)	Hatha-Yoga + compassion meditation program	yes	yes	yes	8	once	75	yes	no treatment
Daukantaite et al., 2018 (42)	A: YOMI B: Yin-Yoga	A: yes; B: yes	A: yes; B: yes	A: yes; B: no	5	twice	A: 60 yoga + 30 PE; B: 60	yes	no treatment
Fischer et al., 2022 (49)	A: Iyengar-Yoga; B: Integrative Yoga (¾ online)	A: yes; B: yes	A: yes; B: no	A: yes; B: no	12	once	90	yes	mindfulness training
Granath et al., 2007 (41)	Not specified	yes	no	no	16	once	NI	yes	cognitive behavioural therapy
Hewett et al., 2018 (46)	Bikram yoga	yes	yes	no	16	three to five times	90	no	no treatment
Hopkins et al., 2016 (48)	Bikram yoga	yes	yes	no	8	twice	90	no	wait-list
Köhn et al., 2013 (43)	Medical standard care + Medical Yoga	yes	yes	yes	12	once	60	no	medical standard care
Maddux et al., 2017 (44)	Power-Yoga	yes	yes	no	8/16	twice	60	no	WaitCross^1^
Michalsen et al., 2012 (13)	A: moderate Iyengar-Yoga; B: intensified Iyengar-Yoga	A: yes; B: yes	A: no; B: no	A: yes; B: yes	12	A: once; B: twice	90	Yes^a^	wait-list
Smith et al., 2007 (47)	Hatha yoga	yes	yes	yes	10	once	60	no	progressive muscle relaxation
Vardar-Wloka, 2019 (39)	A: moderate Iyengar-Yoga; B: intensified Iyengar-Yoga	A: yes; B: yes	A: no; B: no	A: no; B: no	12	A: once; B: twice	90	no	no treatment
Wadhen & Cartwright, 2021 (45)	Hatha yoga (online)	yes	yes	yes	6	twice or three times	50	yes	wait-list
Wolever et al., 2012 (40)	Viniyoga	yes	yes	unclear	12	once	60	yes	A: mindfulness; B: wait-list

min, minutes; PE, Psychoeducation; YOMI, Yin-Yoga with psychoeducation and mindfulness practice; ^a^encouraged.

The Yoga group received 16-week intervention; ^1^WaitCross group did not receive yoga for eight weeks (i.e., passive control group), then yoga practice for eight consecutive weeks. In the meta-analyses, only the 8-week assessment point was considered.

The duration of the yoga interventions ranged from 5 to 16 weeks (median: twelve weeks) with a duration of 50 to 90 minutes per session (median: 67.5 minutes). Yoga was practised once a week in six studies (38, 40, 41, 43, 47, 49) and twice a week in three studies (42, 44, 48). In two studies, one intervention group practised yoga once a week and the other intervention group twice a week (13, 39). Yoga was practised two or three times a week in one study (45) and three to five times a week in another (46). Participants were given homework in six studies (38, 40–42, 45, 49) and encouragement to practice at home in one study (13). Five studies were three-armed, consisting of two yoga groups (13, 39, 42, 49) and two control groups (40). The remaining eight RCTs had one yoga group (38, 41, 43–48), giving a total of 17 yoga groups in the 13 included studies. Yoga intervention was heterogeneous across the studies. For five yoga groups, yoga intervention consisted of Iyengar yoga (13, 39, 49) and for two groups each of Bikram yoga (46, 48) and Hatha yoga (45, 47). In one study, Hatha yoga was also practised, but in conjunction with a compassion meditation programme (38). One group each practiced Yin yoga (42), Viniyoga (40), Power yoga (44) and Medical yoga derived from Kundalini yoga together with medical standard care (43). One study did not specify the yoga tradition used, but included movements from Kundalini yoga (41). One of the intervention groups was a combination of yoga and psychoeducation and therefore was not included in the meta-analysis (42). One intervention group practised integrative yoga, which combined meditation, breathing and relaxation techniques, yoga postures and ethical/philosophical aspects of yoga (49). All 17 intervention groups included yoga postures in their yoga curriculum. Eleven groups also used breathing exercises (38, 40, 42–49) and eight group used meditation (13, 38, 42, 43, 45, 47, 49). The use of meditation in one of the studies is unclear, as the study only reported on the use of mental techniques (40). The three elements of yoga – postures, breathing and meditation – were included in six intervention groups (38, 42, 43, 45, 47, 49).

Nine of the included 13 RCTs compared yoga with a passive control group (13, 38, 39, 42–46, 48) and three studies with an active control group (41, 47, 49). Another study was conducted as a three-armed study with an active and a passive control arm (40). For the passive control, four RCTs had no treatment (38, 39, 42, 46), three used a waitlist control (13, 45, 48) and one passive control group each had medical standard care (43) and a wait-cross condition (eight weeks on a waitlist followed by eight weeks of yoga) (44). The active control groups used mindfulness training (40, 49), progressive muscle relaxation (47) and cognitive behavioural therapy (41). The duration, frequency and programme length of the active control groups were matched to the yoga intervention.

#### 3.2.3 Outcomes measures

In 10 out of 13 studies, stress was assessed by the Perceived Stress Scale (PSS) developed by Cohen et al. The PSS is a 14-item questionnaire that measures the extent to which life situations are perceived as stressful (50). The PSS-14 was been used by five studies (13, 39, 41, 43, 45). The 10-item PSS-10 is a validated, shortened version of the original PSS-14 (51) and was used to assess perceived stress in five studies (40, 42, 44, 46, 49). One study each used the Perceived Stress Questionnaire (PSQ) (48), Lipp’s Stress Symptoms Inventory for Adults (LSSI) (38) and General Health Questionnaire 12-Items (GHQ-12) (47). Quality of life was assessed in eight RCTs using a questionnaire (13, 41, 43–47, 49), mostly the 36-item Short Form Health Survey (SF-36) (13, 46, 47, 49) but also the Quality of Life Inventory (QOLI) (41), the Warwick-Edinburgh Mental Wellbeing Scale (WEMWBS) (45), the Euro Quality of Life Visual Analogue Scale (EQ-VAS) (43), Life Satisfaction measured by three questions on a 7-point-likert scale (44) and the Harmony in Life Scale (HILS) (44). Eight studies used physiological measures to assess stress (38–43, 47, 48). Measurements included salivary cortisol levels (38, 39, 41, 48) and other cortisol-related measures (42), heart rate (39, 41, 43) and heart rate variability (39, 40), blood pressure (39, 41, 43, 47), breathing rate (40) and urinary catecholamines (41). Only three studies reported on safety by reporting of adverse events (13, 39, 49). No adverse events occurred in any of these studies.

#### 3.2.4 Risk of bias in individual studies

**Figure 2:**
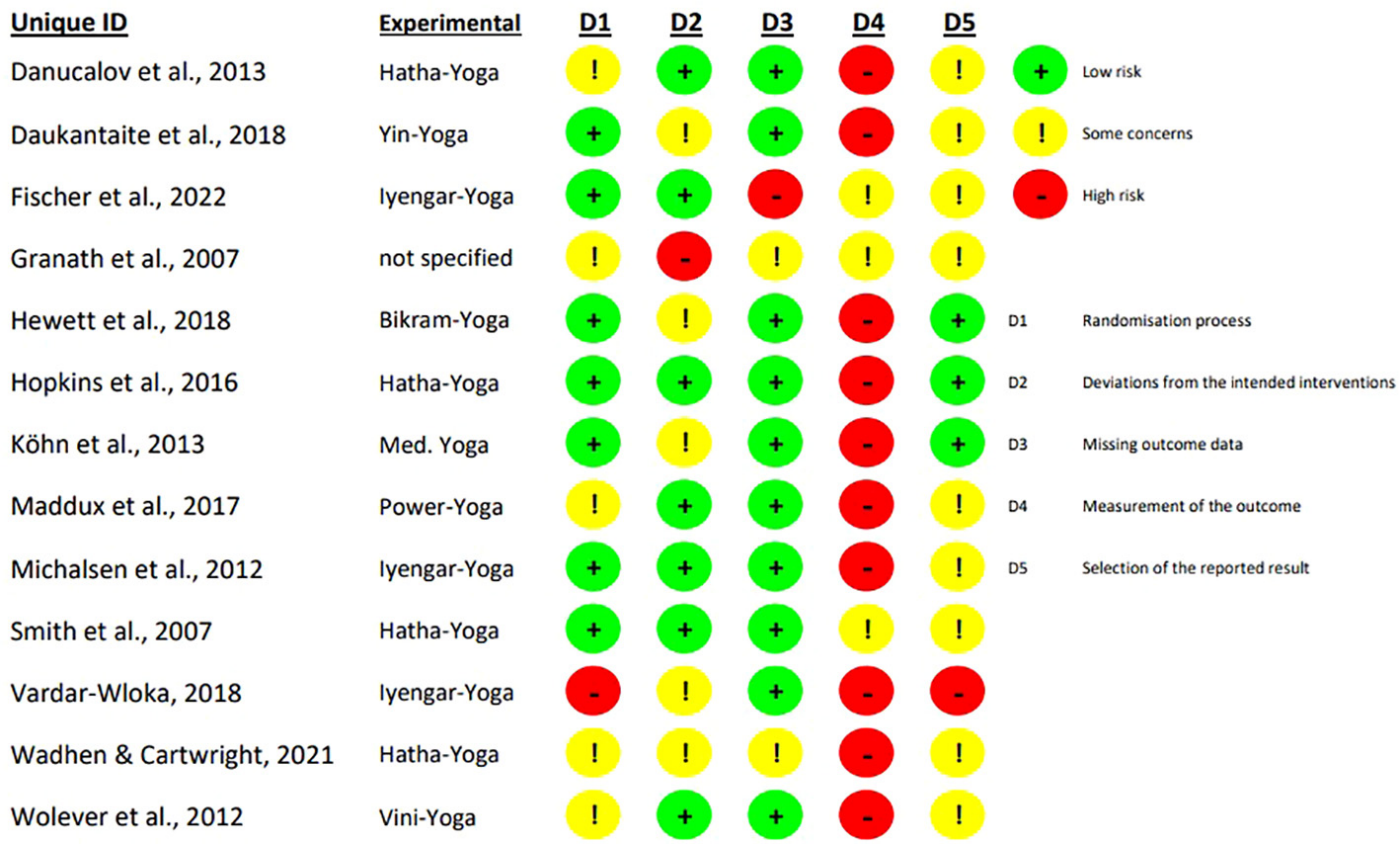
Review authors’ judgements about each risk of bias domain for each included study.

**Figure 3:**
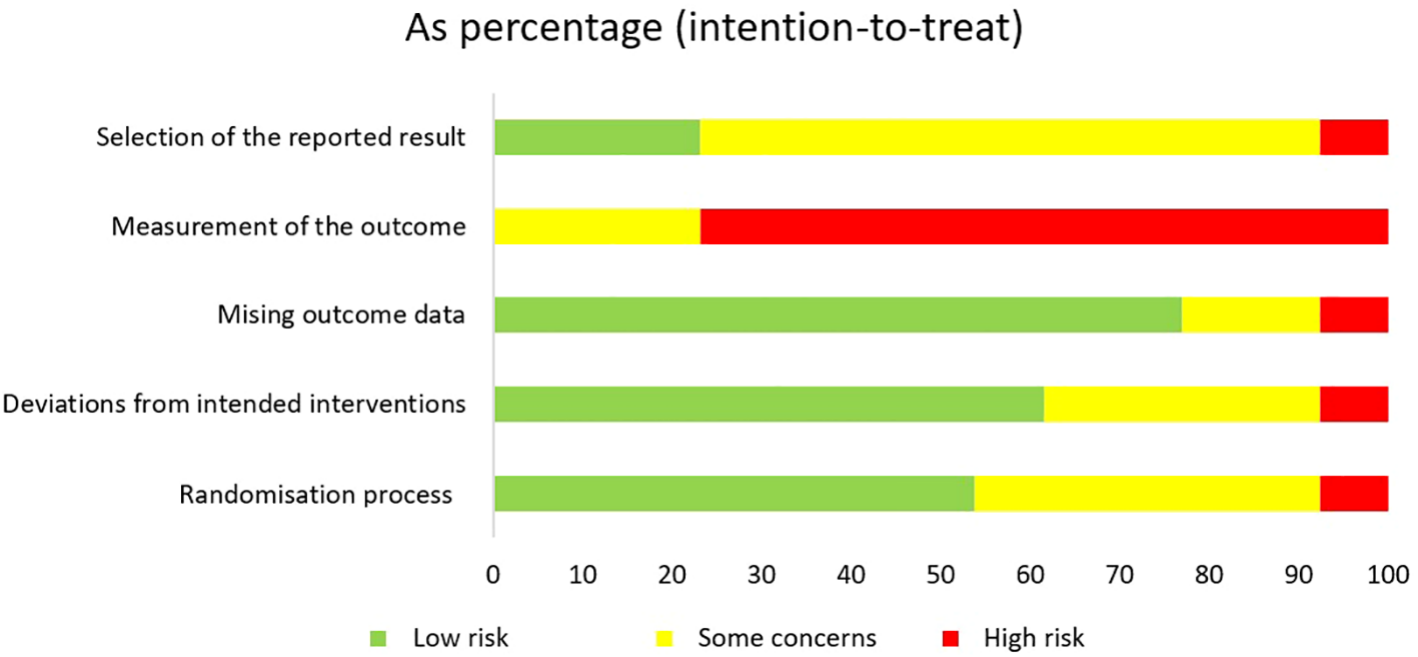
Review authors’ judgements about each risk of bias domain for each included study as percentage.

The assessed risk of bias for the 13 included studies is shown in absolute terms in **Figure 2** and as percentage in **Figure 3**.

All RCTs had a high risk of bias or some concerns in at least one domain. The randomisation process was reported to be adequate in seven studies (13, 42, 43, 46–49). In one study the risk of bias in this domain was judged to be high due to lack of concealed allocation (39). For the derivation of the intended interventions, seven studies had a low risk of bias in domain two (13, 38, 40, 44, 47–49). Five studies did not analyse the effect of assignment leading to some concerns for risk of bias (39, 42, 43, 45, 46). Another study also did not adequately analyse the effect of assignment and in addition, no potential for a substantial effect was reported, resulting in a high risk of bias (41). Two articles had more than 10% missing data and therefore raised some concerns for missing outcome data (41, 45). One study also had more than 10% missing data, but these were intervention-related dropouts (e.g., two participants did not think the programme was effective) (49). Therefore, there is a high risk for the results being biased by missing outcome data. All of the RCTs had a high risk or some concerns for outcome measures. Risk of bias is of some concerns in studies that compared yoga with an active control group (41, 47, 49) and high for studies with passive control (13, 38–40, 42–46, 48). For nine articles no registration number could be found, so the selection of reported outcomes could not be assessed by comparing the prespecified analysis plan with the analysis performed, leading to some concerns about risk of bias (13, 38, 40–42, 44, 45, 47, 49). One article did not analyse its data according to the prespecified analysis and was therefore considered to have a high risk of bias (39).

### 3.3 Analyses of overall effects

#### 3.3.1 Effect on primary outcome: perceived stress

**Figure 4:**
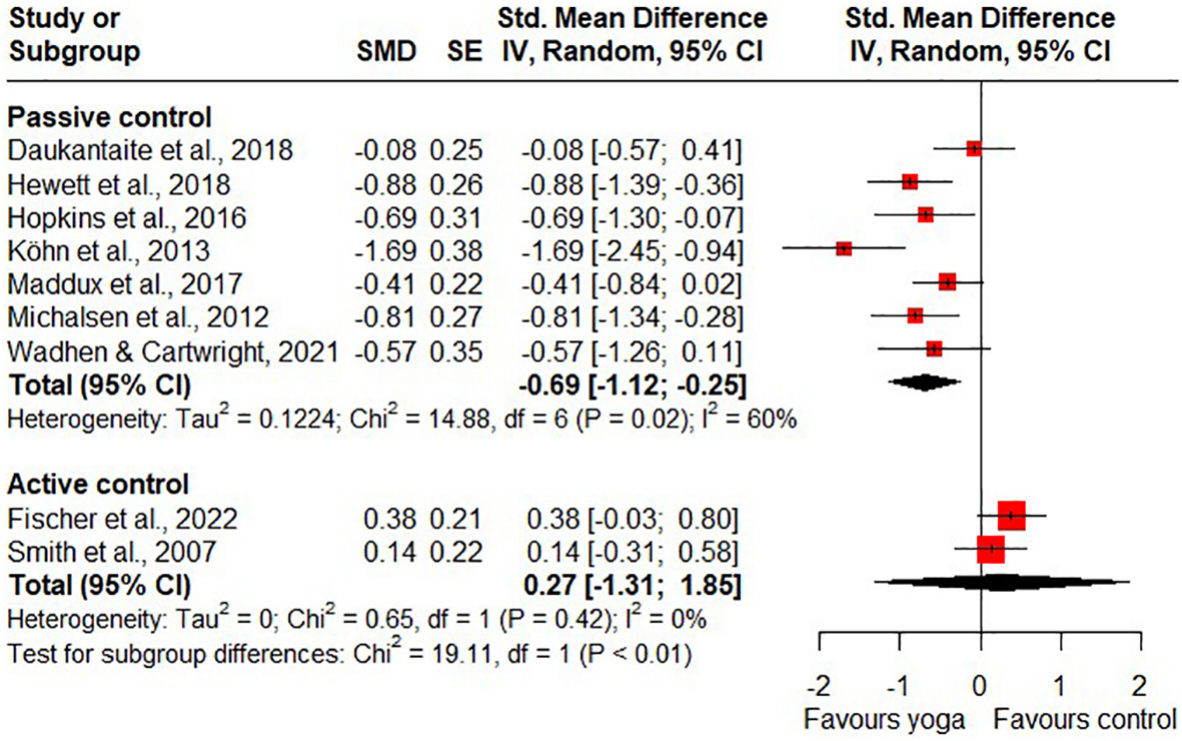
Short-term effects of yoga on perceived stress.

The meta-analysis found a statistically significant difference in short-term effects on perceived stress for yoga compared to passive control groups (SMD = - 0.69, 95% CI -1.12 to -0.25; 7 RCTs; **Figure 4**). The statistical heterogeneity of the included studies with passive control groups was substantial (I^2^ = 60%). Due to the inconsistency and the high likelihood of bias, the quality of the evidence was rated as low. The RCT by Wolever et al. not included in the meta-analysis also reported a statistically significant difference in short-term effects on stress for yoga compared to the passive control group (40). A meta-analysis of the long-term effects of yoga on stress compared to passive control groups was not conducted because the number of eligible studies reporting long-term effects was too small. Only one RCT reported on the long-term effects of yoga on perceived stress compared to a passive control group and found no statistically significant long-term effects on stress (46).

**Figure 5:**
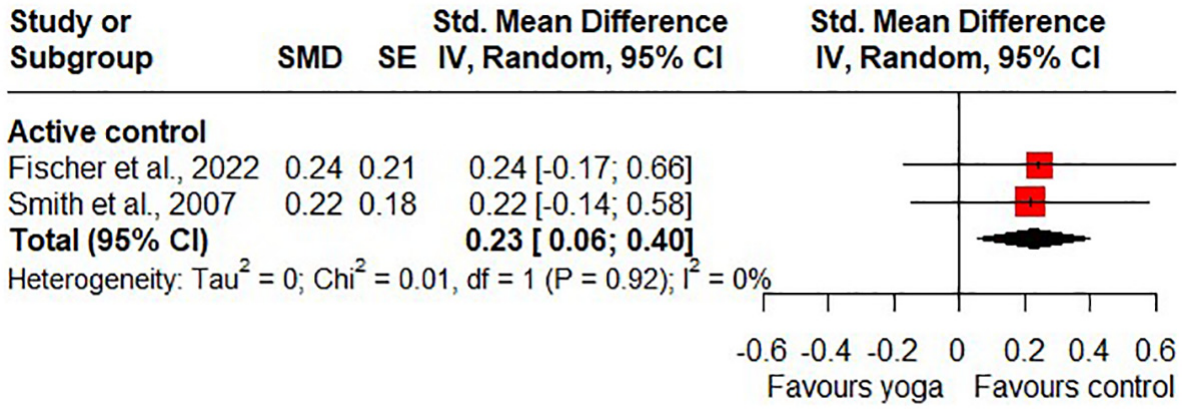
Long-term effects of yoga on perceived stress.

Compared to active control groups, the meta-analysis found no statistically significant differences in short-term effects on stress (SMD = 0.27, 95% CI -1.31 to 1.85; 2 RCTs; **Figure 4**) and statistical heterogeneity was insignificant (I^2^ = 0%). Because of the wide confidence interval, the imprecision of the results was considered to be very serious. In addition, the risk of bias was considered to be high, so the quality of the evidence was downgraded to very low. For long-term effects on stress, the meta-analysis found statistical significance in favour of the active control groups (SMD = 0.23, 95% CI 0.06 to 0.40; 2 RCTs; heterogeneity: I^2^ = 0%; **Figure 5**). The quality of the evidence was rated as low due to the high likelihood of bias and serious imprecision of the results.

#### 3.3.2 Effect on secondary outcome: quality of life

**Figure 6:**
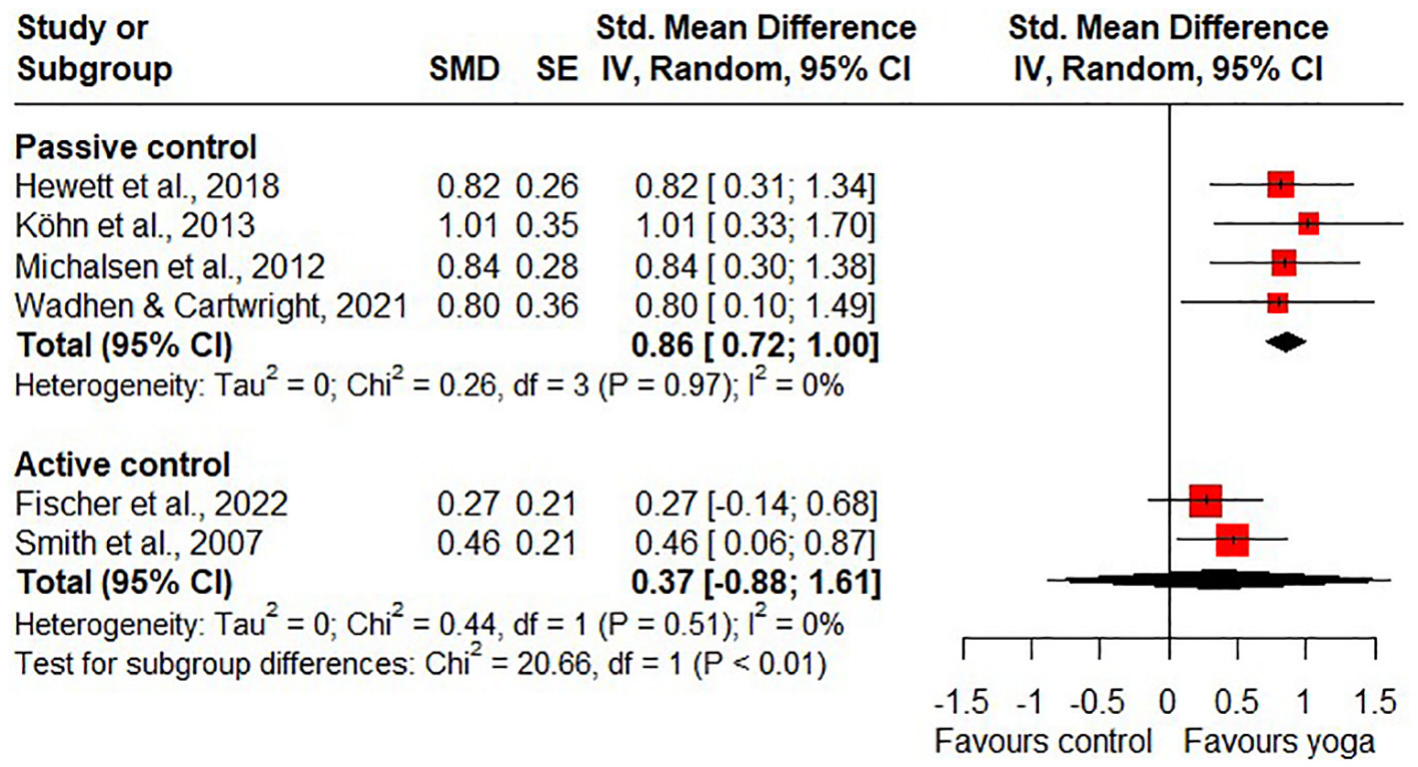
Short-term effects of yoga on quality of life.

Short-term effects on quality of life were found to be statistically significant in the meta-analysis for yoga compared to passive control groups (SMD = 0.86, 95% CI 0.72 to 1.00; 4 RCTs; heterogeneity: I^2^ = 0%; **Figure 6**). The quality of the evidence was rated as moderate because of the high likelihood of bias. Only one RCT reported long-term effects of yoga on quality of life compared to a passive control group and showed no statistically significant long-term effects on stress (46).

**Figure 7:**
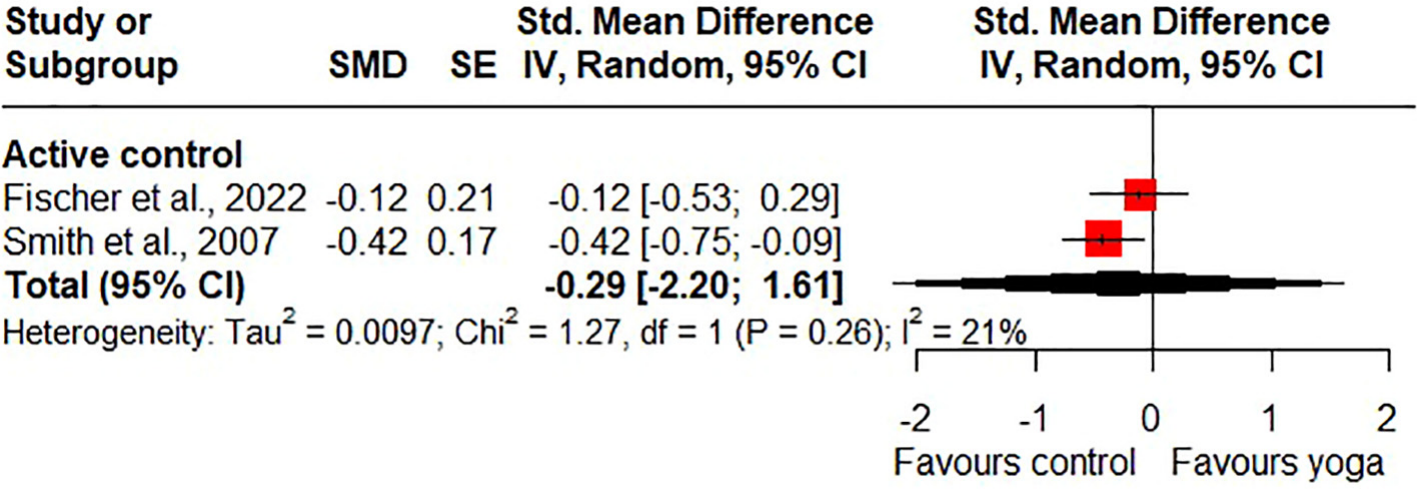
Long-term effects of yoga on quality of life.

Compared to active control groups, there were no statistically significant short-term effects (SMD = 0.37, 95% CI -0.88 to 1.61; 2 RCTs; heterogeneity: I^2^ = 0%; **Figure 6**) and no statistically significant long-term effects on quality of life (SMD = -0.29, 95% CI -2.20 to 1.61; 2 RCTs; heterogeneity: I^2^ = 21%; **Figure 7**). However, one study shows a statistically significant short-term effect on quality of life in favour of yoga and a statistically significant long-term effect on quality of life in favour of the control group (47). Due to the high likelihood of bias and the imprecision of the results, the quality of evidence for the short- and long-term effects of yoga on quality of life compared to active control groups was downgraded to very low.

#### 3.3.3 Effect on secondary outcome: stress-related physiological outcomes

Because of the small number of RCTs reporting physiological outcomes and their variability of the assessment methods, no meta-analysis was carried out. However, three RCTs reported statistically significant short-term effects of yoga on stress-related physiological outcomes compared to passive control groups (40, 42, 48). But it has to be noted that the positive statistically significant effect in the study of Hopkins et al. was only seen in women who already had cortisol reactivity at baseline (48). The studies did not report statistically significant long-term effects of yoga on stress-related physiological outcomes.

#### 3.3.4 Subgroup analyses

No relevant subgroups were identified and therefore no subgroup analysis could be performed.

#### 3.3.5 Sensitivity analyses

As all but one study were judged to be at high risk of bias in at least one domain, no sensitivity analysis was performed due to insufficient data (47).

#### 3.3.6 Risk of bias across studies

Since less than ten studies were included in each meta-analysis, funnel plots were not created.

## 4 Discussion

### 4.1 Summary of evidence

For stress, the meta-analysis of seven studies found low quality evidence for statistically significant short-term effects of yoga compared to passive control groups (wait-list, no treatment). Compared to active control groups (mindfulness training, progressive muscle relaxation), the meta-analysis of two studies found low quality of evidence for a not statistically significant short-term effect of yoga on stress. However, the meta-analysis found low quality evidence for statistically significant long-term effects on stress in favour of the active control groups (mindfulness training, progressive muscle relaxation). It should be noted, that when the GRADE method is used in meta-analyses of non-pharmacological interventions, low or very low quality evidence is often found for stress (52). For quality of life, the meta-analysis of four studies found moderate quality evidence for statistically significant short-term effects of yoga compared to passive control groups. Compared to active control groups, the meta-analyses of two studies found no statistically significant short-term or long-term effects of yoga on quality of life. The quality of evidence for these non-significant effects was very low. Three RCTs reported, that stress-related physiological outcomes were statistically significantly reduced after the intervention compared to a passive control group (short-term effect) (40, 42, 48). No occurrence of adverse events was reported. However, adverse events were only reported in three studies, so the validity of this systematic review in terms of safety is limited (13, 39, 49).

### 4.2 Comparison with prior systematic reviews

The results of this systematic review are in line with the results of previous systematic reviews that investigated the effects of yoga on perceived stress. However, a systematic review and meta-analysis specifically on yoga for stressed adults from the general population was not available. Thus, in his systematic review from 2014, Manoj Sharma included studies that examined the effect of yoga on stress and found that yoga had a positive effect on stress (53). Unlike our systematic review, the participants were not all adults and they were not stressed. In addition, the origin of the included studies was homogeneous (ten of the eleven studies were from the United States), which limits the applicability of the results to other geographical regions. The systematic review by Chong et al., published in 2011, included studies that investigated the effect of yoga on stress in healthy adults and concluded that yoga was effective in reducing stress (23). However, Chong et al. included healthy and non-stressed adults and did not conduct a meta-analysis. Another systematic review by Wang et al. in 2020 that examined the effects of yoga on stress, also included healthy and non-stressed adults and did not perform a meta-analysis (7). More studies than in the review of Chong et al. were included, but with a very heterogeneous intervention duration of four to 28 weeks and reported methodological problems in the included studies. They revealed that yoga has positive effects on stress reduction in healthy adults. Della Valle et al. conducted a meta-analysis and included studies that examined the effect of yoga on stress in stressed employees compared to passive control groups (24). The meta-analysis, published in 2020, showed that workplace yoga had a statistically significant positive effect on employees’ perceived stress compared to passive control groups. The risk of bias was also considered to be similar to that of the current systematic review. However, the systematic review by Della Valle et al. included only six randomised and also non-randomised controlled trials on employees and yoga in the workplace.

### 4.3 External and internal validity

There is heterogeneity in the approaches used to measure stress in the included studies. Stress was mainly measured with the PSS-10 or PSS-14, but also with the LSSI, PSQ and GHQ-12. These validated and reliable self-reported questionnaires are commonly used in clinical settings. The PSS-10 and PSS-14 assess the extent to which people feel their lives are unpredictable, uncontrollable, and overwhelming, rather than focusing on specific events (54). In contrast, the LSSI assesses objective stressful situations (55). The PSQ also considers the positive dimension joy (56). One included study used the GHQ-12 as an outcome measure for stress (47). The GHQ-12 is a psychological distress questionnaire, with high scores indicating poorer mental health. It is not a validated perceived stress questionnaire (57). Excluding this study would not impact the results. Physiological measurements were mainly cortisol, blood pressure and heart rate. This heterogeneity in the measurement approaches and in the selection of the questionnaires reflects the different mechanisms and effects of stress on an individual.

In addition, the participants in the included studies who were assessed as stressed were recruited from the general population or were employees of companies, so the results of this systematic review can be representative for stressed adults in the general population. However, the applicability of the results is limited by the fact that more than half of the studies included in the meta-analyses were conducted in Germany and Sweden. With a median of 91.5% female participants, women were over-represented in these studies, which limits their applicability to both sexes.

Furthermore, risk of bias was only assessed in individual domains, and no overall rating was reported. Overall risk of bias represents the highest rated subdomain. For measurement of the outcome, all studies were rated as high or some concerns, because participants reported their perceived stress using a self-report measure and could not be blinded. Therefore, it cannot be excluded that participants’ expectations of the yoga interventions may have influenced the reported outcome. According to the risk of bias assessment tool, it is assumed that the participants’ expectations of the yoga and active control interventions are similar, and therefore the risk of bias in the measurement of the outcomes in studies comparing yoga with an active control group was rated as having only some concerns (41, 47, 49). A sensitivity analysis was not performed because of the small number of included studies, which further limits the power of the meta-analyses due to the lack of robustness and reliability checks. To draw conclusions about the effects of yoga on stress in stressed adults from the general population, more studies with a low risk of bias are needed.

### 4.4 Strengths and limitations

To the best of our knowledge, this is the first systematic review and meta-analysis of yoga on stress for stressed adults from the general population. The systematic review was conducted according to a pre-registered protocol, taking into account the current Cochrane guidelines for systematic reviews. The systematic review has a detailed, systematic and reproducible search strategy with no restrictions on language and type of publication. Inclusion and exclusion criteria were clearly defined and only RCTs were included. Screening of literature, data extraction as well as assessment of risk of bias and quality of evidence each were conducted by two authors independently.

A limitation of this systematic review and meta-analysis is the small number of RCTs included. In addition, it was not possible to account for and quantify adherence rates in the intervention and control groups based on the information in the included studies. It was also not possible to perform a subgroup analysis, because the types of yoga, as well as the frequency and duration were very heterogeneous across the trials. However, all types of yoga included more than one component (postures, breathing, meditation). Yoga heterogeneity is a common problem in yoga meta-analyses, but a systematic review of 306 yoga RCTs found that different styles of yoga did not differ in the likelihood of reaching positive conclusions (58). Outcome measures and their assessment time points were also heterogeneous. Therefore, a meta-analysis for physiological outcomes could not be performed on the basis of the included studies. It should be noted that heterogeneity is typical for stress, as stress is highly subjective and “the field is characterized by diversity in the definition and measurement of stress” (59). In order to more appropriately account for uncertainty given the small number of included studies and their heterogeneity, the Knapp-Hartung small sample correction was used to pool treatment effects (33). Nevertheless, the evidence from RCTs comparing yoga interventions with passive control groups is limited by substantial heterogeneity. The number of studies comparing yoga with active control groups included in the meta-analysis was very small, which severely limits the interpretation of these results. None of these active control groups included types of physical activities. Additionally, most studies had a high risk of bias regarding outcome assessment. Risk of bias in the selection of the reported outcome was also very often rated as some concerns. Adverse events were not reported in most of the included studies, so it is not possible to make a clear statement about safety. A prior systematic review of adverse events associated with yoga practice found that most adverse events were mild, transient and often related to a medical precondition, so there is no reason to discourage yoga for healthy adults (60).

### 4.5 Implications for further research

Due to the low methodological quality and heterogeneity of the included studies, the interpretation of the evidence found in the meta-analyses is limited. Therefore, future studies should focus their attention on ensuring rigorous methodology and reporting, selecting an appropriate sample size, using an adequate randomisation process (e.g. centrally managed or remotely controlled method) and reporting this accurately, and analysing data by an intention-to-treat analysis. Studies should be registered and should not deviate from the procedure described in the published protocol. In addition, reasons for dropout should be documented, missing outcome data should be sufficiently small, and analysis methods and sensitivity analyses should ensure that the evidence for the results was not biased by the missing outcome data (61).

Further studies of yoga should define the intervention in terms of intensity, duration and frequency of yoga sessions, explain the rationale for the choice of yoga style, and report on the training of the yoga teacher and adherence to the sessions (62). In addition, relevant and valid outcome measures, preferably objective and subjective criteria, should be selected. As blinding of participants is not possible, potential bias should be minimised by having the results assessed by independent assessors.

Furthermore, future studies should compare yoga to active control groups. Several options for appropriate active control groups include an attention control group, in which participants receive similar attention and interaction as the yoga group. This could help to separate the specific effects of yoga from the benefits associated with engagement. Another option is to use practices that include an element of yoga, such as movement, breathing exercises or relaxation. This could be a way to explore whether the holistic combination of these elements is more beneficial than their isolated application. A comparison of yoga with an established, evidence-based treatment as a control group is also recommended. In this case, when choosing an appropriate control group, researchers must to decide whether they are interested in demonstrating non-inferiority of yoga (i.e., whether yoga is not significantly worse than the established, evidence-based treatment), or whether they want to prove that yoga may offer greater benefits.

The assessment of long-term effects should also be considered in future research in order to provide a more comprehensive assessment of the effect of yoga on stress or quality of life. This is particularly important as Smith et al. found that yoga had a statistically significant short-term effect on quality of life compared to the active control group consisting of progressive muscle relaxation, but the active control group had showed a statistically significant long-term effect on quality of life compared to yoga (47).

## 5 Conclusion

The meta-analyses found a low quality evidence for statistically significant short-term effects of yoga on stress and moderate quality evidence for statistically significant long-term effects of yoga on quality of life in stressed adults from the general population compared to passive control groups. Compared to active control groups, the meta-analysis found low quality evidence for statistically significant long-term effects on stress in favour of active controls. Although the methodological quality and heterogeneity of the included studies limits the interpretability of the results, the number of studies measuring the long-term effects is small, and it was not possible to make a clear statement about safety, yoga can be recommended for stressed adults from the general population.

## Data Availability

The original contributions presented in the study are included in the article/**Supplementary Material**. Further inquiries can be directed to the corresponding author.
