# Supplementary material for: Effects of yoga on stress in stressed adults: a systematic review and meta-analysis
Source: Front Psychiatry. 2024 Nov 1;15:1437902. doi: 10.3389/fpsyt.2024.1437902 (PMC11563964; doi:10.3389/fpsyt.2024.1437902)
Supplement: Supplementary file 1 [file DataSheet1.pdf]

PubMed:

("Yoga"[Mesh] OR yoga*[Title/Abstract] OR yogi*[Title/Abstract] OR asana*[Title/Abstract] OR pranayama [Title/Abstract] OR dhyana [Title/Abstract] OR dharana [Title/Abstract] OR "Surya Namaskar*" [Title/Abstract]) AND (stress*[Title/Abstract] OR "Stress, Psychological"[Mesh] OR "Stress, Physiological"[Mesh] OR "Occupational Stress"[Mesh] OR "Psychological Distress" [Mesh] OR "Financial Stress"[Mesh])

Filter: RCT, Clinical Trial

Cochrane:

((Stress, Psychological.ti,ab,kw,sh) OR (Stress, Physiological.ti,ab,kw,sh) OR (Occupational Stress.ti,ab,kw,sh) OR (Psychological Distress.ti,ab,kw,sh) OR (Financial Stress.ti,ab,kw,sh) OR (stress*.ti,ab,kw)) AND ((yoga*.ti,ab,kw,sh) OR (yoga*.ti,ab,kw) OR (yogi*.ti,ab,kw) OR (asana*.ti,ab,kw) OR (pranayama.ti,ab,kw) OR ("Surya Namaskar*".ti,ab,kw) OR (dhyana.ti,ab,kw) OR (dharana. ti,ab,kw ))

Scopus:

TITLE-ABS-KEY(*yoga) OR TITLE-ABS-KEY(yoga*) OR TITLE-ABS-KEY(yogi*) OR TITLE-ABS-KEY(asana*) OR TITLE-ABS-KEY(pranayama) OR TITLE-ABS-KEY(dhyana) OR TITLE-ABS-KEY(dharana) OR TITLE-ABS-KEY("surya namaskar*") AND TITLE-ABS-KEY (stress) AND NOT TITLE-ABS-KEY (animal OR tissue OR mice OR cancer OR review OR observational OR "case report" OR "case study" OR parkinson OR sclerosis OR *arthritis OR cardiac OR infant OR "chronic pain" OR "back pain" OR hyperten* OR cell OR PTSD OR "anxiety disorder" OR "qualitative study" OR "depressive disorder" OR "major depression" OR "irritable bowel") AND ( LIMIT-TO ( SRCTYPE,"j" ) ) AND ( LIMIT-TO ( DOCTYPE,"ar" ) )

PsyInfo:

(Yoga* OR yogi* OR asana* OR pranayama OR dhyana OR dharana OR “surya namaskar*”) AND stress*
Filter: Boolean/Phrase, Apply equivalent subjects, Peer Reviewed Journal, Fully published, Adulthood, Human
literature review, meta analysis, systematic review

Base:
yoga and stress

**Supplementary Data 1**: Search strategy for systematic literature search

**Summary of findings:**

## Yoga compared to passive control groups for stress

**Patient or population:** stress
**Setting:**
**Intervention:** Yoga
**Comparison:** passive control groups

| Outcomes | Anticipated absolute effects* (95% CI) | | Relative effect (95% CI) | № of participants (studies) | Certainty of the evidence (GRADE) | Comments |
|---|---|---|---|---|---|---|
| | **Risk with passive control groups** | **Risk with Yoga** | | | | |
| Stress | - | SMD **0.68 SD lower** (1.12 lower to 0.25 lower) | - | 401 (7 RCTs) | ⊕⊕◯◯ Low[a,b,c,d] | |
| Quality of life | - | SMD **0.86 SD higher** (0.72 higher to 1 higher) | - | 206 (4 RCTs) | ⊕⊕⊕◯ Moderate[a,e,f] | |

***The risk in the intervention group** (and its 95% confidence interval) is based on the assumed risk in the comparison group and the **relative effect** of the intervention (and its 95% CI).

**CI:** confidence interval; **SMD:** standardised mean difference

**GRADE Working Group grades of evidence**
**High certainty:** we are very confident that the true effect lies close to that of the estimate of the effect.
**Moderate certainty:** we are moderately confident in the effect estimate: the true effect is likely to be close to the estimate of the effect, but there is a possibility that it is substantially different.
**Low certainty:** our confidence in the effect estimate is limited: the true effect may be substantially different from the estimate of the effect.
**Very low certainty:** we have very little confidence in the effect estimate: the true effect is likely to be substantially different from the estimate of effect.

**Explanations**

a. Risk of bias: high risk of bias in measurement of the outcome
b. Inconsistency: $I^2$ = 60 %
c. Indirectness: mostly women, there are a lot of different yoga-types but so is yoga, perceived stress is selfreported but stress is subjective
d. Imprecision: adequate sample size, not wide CI
e. Inconsistency: $I^2$ = 0
f. Indirectness: mostly women, there are a lot of different yoga-types but so is yoga, Quality of life is selfreported but QoL is subjective

**Supplementary Figure 1:** GRADE rating of evidence (yoga vs. passive control)

**Summary of findings:**

## Yoga compared to active control group for stress

**Patient or population:** stress
**Setting:**
**Intervention:** Yoga
**Comparison:** active control group

| Outcomes | Anticipated absolute effects* (95% CI) | | Relative effect (95% CI) | № of participants (studies) | Certainty of the evidence (GRADE) | Comments |
|---|---|---|---|---|---|---|
| | Risk with active control group | Risk with Yoga | | | | |
| Stress short-term | - | SMD **0.27 SD higher** (1.31 lower to 1.85 higher) | - | 233 (2 RCTs) | ⊕◯◯◯ Very low[a,b,c,d] | |
| Quality of life short-term | - | SMD **0.37 SD higher** (0.88 lower to 1.61 higher) | - | 233 (2 RCTs) | ⊕◯◯◯ Very low[a,b,c,d] | |
| Stress- long-term | - | SMD **0.23 SD higher** (0.06 higher to 0.4 higher) | - | 233 (2 RCTs) | ⊕⊕◯◯ Low[a,b,c,e] | |
| Quality of life long-term | - | SMD **0.29 SD lower** (2.2 lower to 1.61 higher) | - | 233 (2 RCTs) | ⊕◯◯◯ Very low[a,b,c,d] | |

***The risk in the intervention group** (and its 95% confidence interval) is based on the assumed risk in the comparison group and the **relative effect** of the intervention (and its 95% CI).

**CI:** confidence interval; **SMD:** standardised mean difference

**GRADE Working Group grades of evidence**
**High certainty:** we are very confident that the true effect lies close to that of the estimate of the effect.
**Moderate certainty:** we are moderately confident in the effect estimate: the true effect is likely to be close to the estimate of the effect, but there is a possibility that it is substantially different.
**Low certainty:** our confidence in the effect estimate is limited: the true effect may be substantially different from the estimate of the effect.
**Very low certainty:** we have very little confidence in the effect estimate: the true effect is likely to be substantially different from the estimate of effect.

**Explanations**

a. Risk of bias: some concerns with the selection of the reported result and one study had more than 10 % missing data (high risk of bias because the drop-outs were intervention-related)
b. Inconsistency: $I^2 = 0$
c. Indirectness: mostly women, there are a lot of different yoga-types but so is yoga
d. Imprecision: relatively large sample size, widely CI
e. Imprecision: relatively large sample size, not widely CI

**Supplementary Figure 2**: GRADE rating of evidence (yoga vs. active control)
